# Sarcoidosis: An Old but Always Challenging Disease

**DOI:** 10.3390/diagnostics11040696

**Published:** 2021-04-14

**Authors:** Claudio Tana

**Affiliations:** COVID-19 Medicine Unit and Geriatrics Clinic, SS Annunziata Hospital of Chieti, 66100 Chieti, Italy; claudio.tana@asl2abruzzo.it; Tel.: +39-0871-357905; Fax: 0871-357905

Sarcoidosis is a granulomatous disease which can involve every organ, and can manifest with nonspecific clinical pictures that can be misdiagnosed with those observed with other disorders [1]. Despite the fact that, for a long time it has been considered as a rare disease, the evolution of modern computed tomography techniques, with the introduction also of deep learning software in the field of artificial intelligent technology, has helped to improve the diagnostic capacity of this uncommon disease [2].

In the COVID-19 era, sarcoidosis is giving a great difficulty of differential diagnosis, especially when there is a predominant lung involvement or the two conditions exist [2,3].

The diagnosis of sarcoidosis can therefore be very challenging and a thorough evaluation, based on a multidisciplinary approach and holistic view, is often useful to achieve the correct diagnosis [4].

A complete pictorial and literature essay aimed at showing the main diagnostic features of pulmonary sarcoidosis versus pneumonia from the SARS-CoV-2 infection is presented herein [1]. Although some findings can be commonly observed in both conditions, there are several imaging features that can be useful to distinguish between the two disorders [1].

Based on the necessity to reveal the typical presence of non-caseating and non-necrotizing granulomas, biopsy is often indicated although it can be invasive, especially in deep areas or those at risk of bleeding and or infection [4].

Due to this often unbalanced cost–benefit ratio, every effort should be made to achieve the diagnosis in a way that is the least invasive, especially when sarcoidosis manifests with typical clinical pictures, such as the Löfgren syndrome (LS), characterized by fever, bilateral hilar lymphadenopathy, acute onset of erythema nodosum and migratory polyarthritis. An invasive approach can be temporary avoided in this situation, as LS is virtually diagnostic of sarcoidosis [4]. 

Lungs are the organs that are predominantly affected, but other districts can be compromised such as skin, eyes, and the gastrointestinal and nervous systems. Laboratory exams in sarcoidosis are often nonspecific, and serum angiotensin converting enzyme (ACE) elevation is neither sensitive nor specific. In their study, Dr. Papasavvas et al. evaluated the usefulness of lysozyme versus ACE and of polyclonal antibody activation as laboratory tests supporting the diagnosis of ocular sarcoidosis. Their results are reported below [5].

Other authors reported an extensive overview of the diagnostic workout of sarcoidosis and methods of follow-up. Some emblematic cases of potential misdiagnosis that can be major clinical problems in daily practice were moreover reported. Several conditions can indeed be misdiagnosed with sarcoidosis, such as infections, neoplasms, immunodeficiencies, and drug-induced diseases [6,7,8]. In this issue, news about management of sarcoidosis and drug treatment are also presented, and an interesting review on the perceived quality of life of patients, an aspect that should not be neglected [2,9,10], is reported here separately. 

We hope that readers will appreciate this issue, which is meant to be an attempt at reporting the last evidence on diagnosis and management of an old but always challenging clinical condition.
